# Sedimentary Sulphur:Iron Ratio Indicates Vivianite Occurrence: A Study from Two Contrasting Freshwater Systems

**DOI:** 10.1371/journal.pone.0143737

**Published:** 2015-11-24

**Authors:** Matthias Rothe, Andreas Kleeberg, Björn Grüneberg, Kurt Friese, Manuel Pérez-Mayo, Michael Hupfer

**Affiliations:** 1 Department of Chemical Analytics and Biogeochemistry, Leibniz-Institute of Freshwater Ecology and Inland Fisheries, Berlin, Germany; 2 Department of Geography, Humboldt-Universität zu Berlin, Berlin, Germany; 3 Department of Freshwater Conservation, Brandenburg University of Technology Cottbus-Senftenberg, Bad Saarow, Germany; 4 UFZ-Department of Lake Research, Helmholtz Centre for Environmental Research, Magdeburg, Germany; 5 Institute of Environmental Physics, University of Bremen, Bremen, Germany; Argonne National Laboratory, UNITED STATES

## Abstract

An increasing number of studies constrain the importance of iron for the long-term retention of phosphorus (P) under anoxic conditions, i.e. the formation of reduced iron phosphate minerals such as vivianite (Fe_3_(PO_4_)_2_⋅8H_2_O). Much remains unknown about vivianite formation, the factors controlling its occurrence, and its relevance for P burial during early sediment diagenesis. To study the occurrence of vivianite and to assess its relevance for P binding, surface sediments of two hydrologically contrasting waters were analysed by heavy-liquid separation and subsequent powder X-ray diffraction. In Lake Arendsee, vivianite was present in deeper sediment horizons and not in the uppermost layers with a sharp transition between vivianite and non-vivianite bearing layers. In contrast, in lowland river Lower Havel vivianite was present in the upper sediment layers and not in deeper horizons with a gradual transition between non-vivianite and vivianite bearing layers. In both waters, vivianite occurrence was accompanied by the presence of pyrite (FeS_2_). Vivianite formation was favoured by an elevated iron availability through a lower degree of sulphidisation and was present at a molar ratio of total sulphur to reactive iron smaller than 1.1, only. A longer lasting burden of sediments by organic matter, i.e. due to eutrophication, favours the release of sulphides, and the formation of insoluble iron sulphides leading to a lack of available iron and to less or no vivianite formation. This weakening in sedimentary P retention, representing a negative feedback mechanism (P release) in terms of water quality, could be partly compensated by harmless Fe amendments.

## Introduction

Phosphorus (P) is one of the key nutrients in aquatic systems and often governs their primary production. Although in many European countries nutrient inputs have been significantly reduced within the last 30 yr, the eutrophication of rivers, lakes and coastal seas is still an issue [1, 2]. Ultimately, it is the balance between sources and sinks which determines P availability for primary producers in aquatic ecosystems. Besides fluvial and groundwater P export, the burial in sediments accounts for the major loss of P in aquatic ecosystems.

The sequestration of P in sediments is the result of multiple chemical reactions which are driven by the mineralisation of organic matter (OM) through microorganisms. Along with these reactions the elemental cycles of iron (Fe) and sulphur (S) play a crucial role in binding of P in sediments. Einsele [3] and Mortimer [4] revealed the tight coupling between the Fe and the P cycle since iron(oxyhydr)oxides act as efficient sorption agents for orthophosphate (PO_4_^3-^). By this mechanism PO_4_^3-^ is trapped and surface sediments can act as a barrier for upward diffusing PO_4_^3-^ as long as the overlying water is oxic [5]. Under anoxic conditions, however, reduction of iron(oxyhydr)oxides may result in the release of PO_4_^3-^ into the water column. Due to this redox sensitivity, P associated with Fe has long been considered not to be a significant burial form in the long-term.

The cycling of S is closely coupled to the transformations of P and Fe. Since free sulphides (S^2-^) react with ferrous Fe (Fe(II)) to form insoluble Fe sulphides (FeS_x_), the decomposition of OM by sulphate reducing microorganisms depletes the reactive Fe pool (this includes iron(oxyhydr)oxides and dissolved ferrous Fe) in the sediment and counteracts the functioning of Fe in binding P. Simultaneously, iron(oxyhydr)oxide-P compounds may undergo reductive dissolution in the presence of S^2-^ which leads to increased PO_4_^3-^ concentrations in the water [6–9]. This reductive phosphate release can also trigger authigenic P mineral formation, in particular reduced Fe phosphates if not all reactive Fe is precipitated by S^2-^. Therefore, the extent of S^2-^ production and the amount of reactive Fe available crucially determines the effectivity of Fe in binding P, significantly influencing P retention in the short- and also in the long-term [10, 11].

Recent studies from fresh- and marine water bodies prove Fe-P compounds to be of significance for P burial in anoxic sediments and suggest that this is due to the formation of stable reduced Fe phosphate minerals [12–19]. However, the determination of the exact binding form(s) is difficult and the mechanisms leading to lasting Fe-associated P burial under anoxic conditions are not fully understood.

Vivianite (Fe_3_(PO_4_)_2_ ⋅ 8H_2_O) is the most common reduced Fe(II)-phosphate mineral forming under anoxic, non-sulphidic conditions in Fe- and organic-rich soils and sediments [8, 20, 21]. The occurrence of vivianite in sediments has been proposed by many authors, however, the direct identification is difficult. There is limited knowledge about its formation conditions and its relevance for P retention in surface sediments. Only recently, we applied a novel approach which enabled us to identify vivianite by X-ray diffraction and showed vivianite to be a significant burial form in a lake sediment artificially enriched in Fe [19].

To investigate the relevance of Fe-associated P burial through the formation of reduced Fe(II)-phosphate minerals, we analysed surface sediments of two contrasting eutrophic freshwater systems, i.e. a deep lake that is poor in Fe (Lake Arendsee), and a shallow lowland river rich in Fe (Lower Havel). In particular, our study aims for a better understanding under which sedimentary conditions vivianite occurs and how the cycling of Fe, P and S affects the formation of vivianite. For these purposes, surface sediments were separated by a heavy-liquid to identify vivianite by X-ray diffraction. Furthermore, sediments were characterised by elemental analysis, P fractionation, total inorganic S extraction and radioisotopic dating.

## Materials and Methods

### Study sites and sample collection

Lake Arendsee is a dimictic, eutrophic karst lake (z_max_ = 48 m, z_mean_ = 29 m, A = 5.13 km^2^) located in northern Germany (52°53′21′′ N, 11°28′27′′ E). It has a long water residence time (50–60 yr, [22]). During summer stratification (April-December) the hypolimnion becomes increasingly anoxic and S^2-^ is present from August to December. The surface sediment has a low Fe content and is rich in Ca and OM (5–15 cm: Fe 3 mg gdw^−1^ and OM 20% dw). However, Fe content drastically increases below ≈ 23 cm sediment depth [23]. In the past, there were several attemps to reduce the P concentration of the lake water, at last in 1995, when calcareous mud from the littoral zone of the lake was used for capping the sediment [24]. Today, this Ca-rich layer is located in 12–15 cm sediment depth.

The Lower Havel is a polymictic lowland river located west of Berlin, Germany (52°27′06′′ N, 13°09′29′′ E). The river comprises of lake-like widenings and is contrasting in its hydrological and biogeochemical conditions in comparison to Lake Arendsee. It stretches about 11 km and water depth varies between 4 m and 8 m with a maximum of 11 m. The hydraulic residence time is on average 19 d (2000–2009) [25]. Most of the time of the year, the water column is well oxygenated, however, oxygen depleted conditions in bottom waters may occur occassionally in summer during periods of low winds and high temperatures. The sediments are organic-rich and have a mud-like texture. The depth of oxygen penetration into the sediment is therefore considered to range a few millimetres only. The sediment is non-sulphidic and the Fe content in the upper sediment is about 10 times higher than in Lake Arendsee.

Undisturbed sediment cores (60 mm and 96 mm in diameter, 30–50 cm long) were collected by a gravity corer (UWITEC) at the deepest site in Lake Arendsee and at 8 m water depth in Lower Havel and sectioned into 10 mm or 20 mm slices. For Lake Arendsee sediment cores were collected in June 2007 for sequential P extraction, in February 2014 for the analysis of vivianite deposits (heavy-liquid separation) and elemental analysis, and in October 2014 for total reducible inorganic S (TRIS) extraction and radioisotopic dating. For Lower Havel, sediment cores were taken in October 2011 for sequential P extraction, in May 2012 for TRIS extraction, in October 2013 for the analysis of vivianite deposits (heavy-liquid separation) and elemental analysis, and in October 2014 for radioisotopic dating.

For all sampling of sediments no specific permission was required. The sampling sites did not involve private or protected areas. The research activities were carried out in cooporation with the relevant regulatory body (Senate of Berlin, and State Agency for Flood Protection and Water Management Saxony-Anhalt (LHW)).

### Analysis

#### Radioisotopic dating

Fresh sediment was freeze-dried in a vertical resolution of 1 cm. Samples were sealed with a gas tight foil and stored for one month in order to receive secular equilibrium between ^222^Rn and its short lived daughter isotopes. The *γ*-spectrometric measurements of ^210^Pb, ^226^Ra, ^214^Bi, ^137^Cs, ^40^K and ^7^Be were carried out using a n-type coaxial Ge detector (CANBERRA). Supported ^210^Pb activities were calculated assuming secular equilibrium between ^226^Ra and ^210^Pb. The constant rate of supply (CRS) model [26] and the constant flux and constant sedimentation model (CF-CS) [27] for ^210^Pb were used to construct a sediment chronology.

#### Chemical analysis

Freeze-dried subsamples of sediment (in the following text referred to as “bulk” sediment) were homogenised in an agate mortar and analysed for total metal concentrations after wet digestion (HCl 36%, HNO_3_ 76%, volumetric ratio 1 : 3) in a high-pressure microwave oven (Gigatherm). Concentrations of Al, Ca, Fe, Mn, Mg, and S were determined by inductively coupled plasma optical emission spectrometry (ICP-OES, iCAP 7000series, Thermo Scientific). The acid extractable Fe, henceforth denoted as “Fe”, represents an upper limit of all reactive Fe phases present in the sediment and does not include Fe bound in silicates which is non-reactive in early diagenesis.

Content of OM was determined as loss on ignition (LOI, 6 h, 450°C). Total P (TP) was determined photometrically by the molybdenum-blue method [28] after sediment combustion (2 h, 550°C) and hot digestion (100°C, 2 M HCl) [29].

Sedimentary P forms of fresh sediment were characterised by a sequential extraction according to [30] with slight modifications [31]. The extraction separates six different P forms: (1) loosely adsorbed P, immediately available P (NH_4_Cl-TP), (2) redox sensitive P, mainly bound to Fe-(hydr)oxides (BD-TP), (3) metal-bound P, mainly associated with Fe- and Al-oxides (NaOH-SRP), (4) organic-bound P (NaOH-NRP), (5) P bound in calcium carbonates and apatite (HCl-TP), and (6) residual P determined after digestion of remaining sediment (Res-P). Synthetic, surface-oxidised vivianite powder (Dr. Paul Lohmann GmbH KG) with a blue appearance [19] and a high-density sample containing naturally born vivianite were also sequentially extracted by schemes of [30] and [32]. Following the SEDEX scheme [32], the first two extraction steps were carried out representing loosely adsorbed P (Ex-P, step I), and iron(oxyhydr)oxide-bound P and vivianite (CDB-P, step II).

From fresh sediment samples three inorganic S fractions were determined by sequential extraction [33]: (1) acid volatile S (AVS), considered to represent Fe monosulphides (mackinawite, troilite and pyrrhotite), (2) chromium reducible S (CRS), attributed to disulphidic S forms (pyrite, marcasite) and (3) elemental S (S^0^) [34]. Sulphide concentration in sediment extracts was determined by polarography (Metrohm). Total S content was determined with a CN analyser (Vario EL). Organic S (S_org_) was then calculated as total S - (AVS + CRS + S^0^). Total Fe content was determined by ICP-OES after wet digestion of dry sediment as described above. The relative contribution of mono- and disulphidic-bound Fe on total Fe was then calculated according to stoichiometry and the S^2-^ concentration analysed in the AVS and CRS extracts. All determinations were performed in duplicate.

#### Heavy-liquid separation of sediment

Fresh sediment samples were freeze-dried and a heavy-liquid separation was applied [19] in vertical resolution of 2 cm. A sodium polytungstate solution (3Na_2_ WO_4_⋅9WO_3_⋅H_2_ O, ABCR Co.) was used to separate the sediment into a low-density (*ρ* ≤ 2.3 g cm^−3^) and a high-density (*ρ* > 2.3 g cm^−3^) sample. Deviating from the procedure described in [19], sediment samples were not sieved prior to the heavy-liquid separation.

High-density samples were analysed for the presence of vivianite, appearing as dark blue sediment concretions using a reflected-light microscope. Mineral composition of high-density samples and bulk sediment samples was characterized by powder X-ray diffraction (XRD) with a Bruker AXS D8 diffractometer equipped with Cu-K_*α*_-radiation and a Sol-X solid state detector. The XRD-pattern were measured between 5 and 60° 2*θ* with a step of 0.05° 2*θ* and an integration time of 9 s. Scanning electron micrographs of sediment concretions were obtained with a FEI Quanta 600FEG field emission environmental scanning electron microscope (FE-ESEM).

To get a rough estimate of the magnitude of vivianite-P to sedimentary TP in relation to other P binding partners, concentrations of Al, Ca, Fe, Mn, Mg and P were analysed in the high-density samples by ICP-OES as described above. The share of vivianite-P to sedimentary TP was calculated using the dry mass and the P content of the high-density sample and comparing it with the original sample mass and the P content prior to heavy-liquid separation [19].

## Results

### Vertical distribution of elements and phosphorus fractions

**Fig 1:**
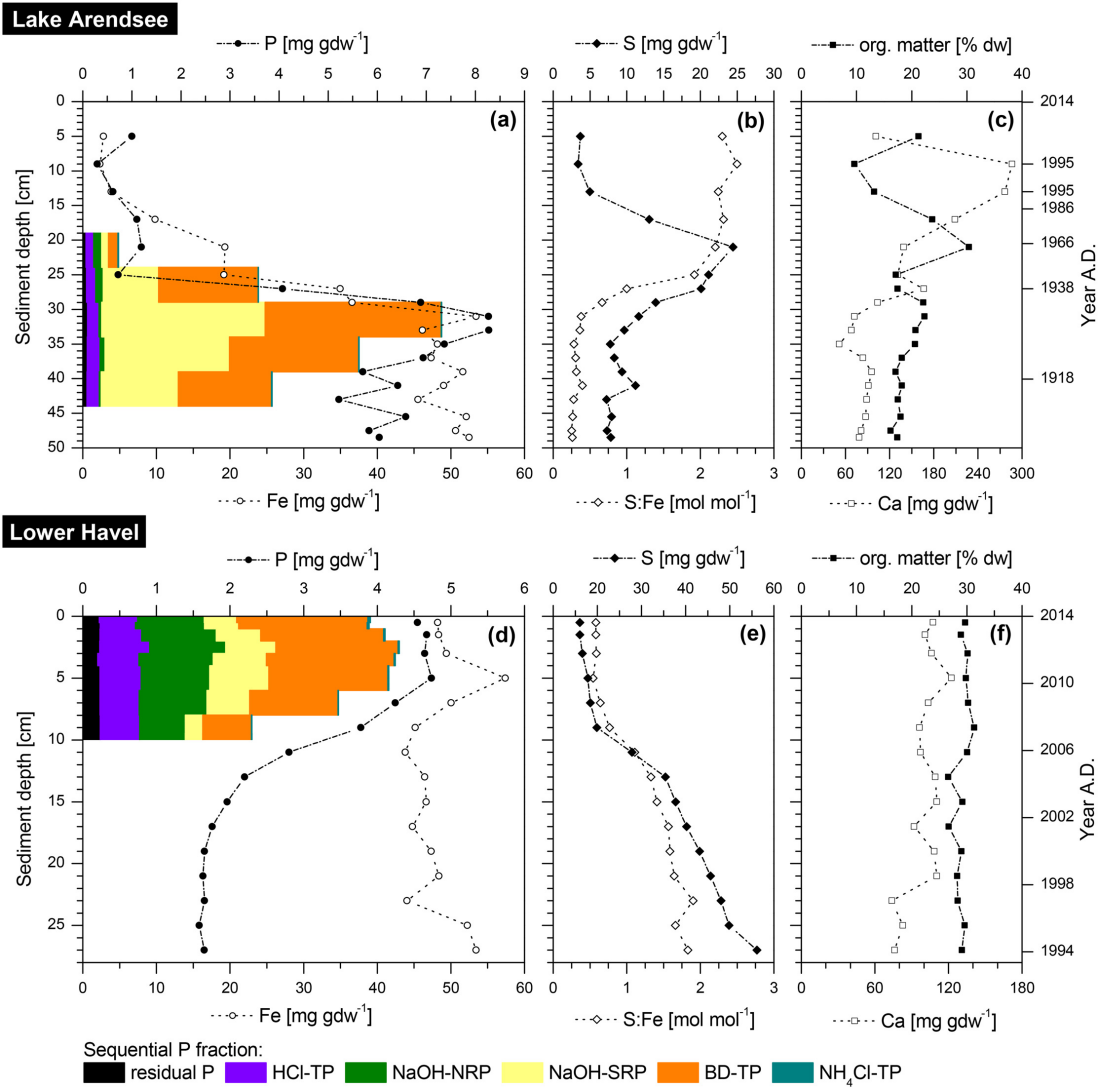
Sediment stratigraphic charts of Lake Arendsee and Lower Havel. **(a, d)** phosphorus (P) and iron (Fe), **(b, e)** molar ratio of total sulphur to reactive iron (S:Fe) and sulphur (S), and **(c, f)** organic matter (org. matter) and calcium (Ca) from February 2014 (Lake Arendsee) and October 2013 (Lower Havel). According to sediment depth the age of the core is indicated. Bar charts in graph **(a)** and **(d)** indicate six different P forms in the correspondent sediment depth layers: (1) NH_4_Cl-TP: loosely adsorbed P, immediately available P, (2) BD-TP: redox sensitive P, mainly bound to Fe-(hydr)oxides, (3) NaOH-SRP: metal P, mainly bound to Fe- and Al-oxides, (4) NaOH-NRP: organic-bound P, (5) HCl-TP: P bound in calcium carbonates and apatite, and (6) Res-P: residual P determined after digestion of remaining sediment. P fractionation data originate from sediment cores taken in June 2007 (Lake Arendsee) and October 2011 (Lower Havel).

For Lake Arendsee and Lower Havel elemental analysis of sediment revealed contrasting trends in the content of sedimentary TP with depth (Fig 1a and 1d). Whereas in sediments of Lake Arendsee TP content was low in the upper 25 cm of the sediment (TP: 0.3–1.2 mg gdw^−1^) and strongly increased further down-core, in sediments of Lower Havel a contrasting trend was evident. Here, the highest TP content was found in the upper 10 cm of the sediment (4.5–4.7 mg gdw^−1^). Further down-core, values decreased by 60% to be as low as 1.7 mg gdw^−1^ in 15 cm depth and were constant to the bottom of the core (27 cm).

For sediments of Lake Arendsee, the depth profile of Fe (reactive, non-silicate Fe) was similar to that of the TP content (Fig 1a). Iron content was low in the upper 25 cm but strongly increased further down-core to be as high as 53 mg gdw^−1^ at 31 cm depth and was constant down to the bottom of the core. For S, there was a peak at 19 cm depth with values to be as high as 25 mg gdw^−1^. The molar ratio of total S to reactive Fe (henceforth denoted as “S:Fe”) showed an opposing vertical trend to that of TP and Fe: in the upper 25 cm the ratio was as high as 2.5 but decreased further down-core to reach values lower than 0.5 (Fig 1b). For Ca and OM content there were large variations in the upper 25 cm of the sediment (Fig 1c). Further down-core, variations were smaller, and Ca averaged to 89 ± 28 mg gdw^−1^ and, OM to 19 ± 2% dw.

For Lower Havel, the depth profile of Fe did not show a decreasing trend as observed for TP. Similar to the content of Ca and OM, Fe content was constant in the upper 27 cm and averaged to 48 ± 4 mg gdw^−1^ (Fig 1d and 1f). The S content increased by more than 300% down-core, with the lowest and highest value to be in the uppermost and lowermost sediment layer (16 mg gdw^−1^ and 56 mg gdw^−1^, respectively). The strongest gradient was between 11–15 cm. Accordingly, the molar S:Fe ratio increased down-core. This ratio was as low aS 0.6 at 5 cm depth and increased to values higher than 1.5 below 17 cm depth (Fig 1e).

Radioisotopic dating of Lake Arendsee revealed a heterogeneous core. Based upon independent time markers, as there was the artificial layer of calcareous mud corresponding to the year 1995, the Chernobyl accident in 1986 and the nuclear weapon tests in the 1960’s, the bottom of the core corresponded to a time prior to 1918 (Fig 1c).

Radioisotopic dating of Lower Havel sediment revealed a homogeneous chronology: the upper 30 cm corresponded to a time span of 22.5 ± 2.5 yr and an avarage dry mass sedimentation rate of 0.144 ± 0.03 g cm^−2^ yr^−1^. Accordingly, an increasing depth represented an increasing age: 5 cm 3.4 ± 0.4 yr, 10 cm 8.5 ± 0.8 yr, 15 cm 10.7 ± 0.8 yr, 20 cm 14.4 ± 0.9 yr, and 25 cm 18.7 ± 2.0 yr (Fig 1f).

P fractionation data from both waters revealed that elevated TP content (Lake Arendsee: 25–44 cm, Lower Havel: 0–10 cm sediment depth) was due to increased burial of two P forms: redox sensitive, Fe-bound P (BD-TP) and metal-bound P (NaOH-SRP) (Fig 1a and 1d). For Lake Arendsee, these two P forms accounted for 80–95% of TP between 25–44 cm sediment depth but for less than 45% between 19–25 cm sediment depth. For Lower Havel, the relative contribution of BD-TP and NaOH-SRP was 40–60% of TP in the upper 10 cm of the sediment and decreased between 6 cm and 10 cm depth.

### Total reducible inorganic sulphur (TRIS)

**Fig 2:**
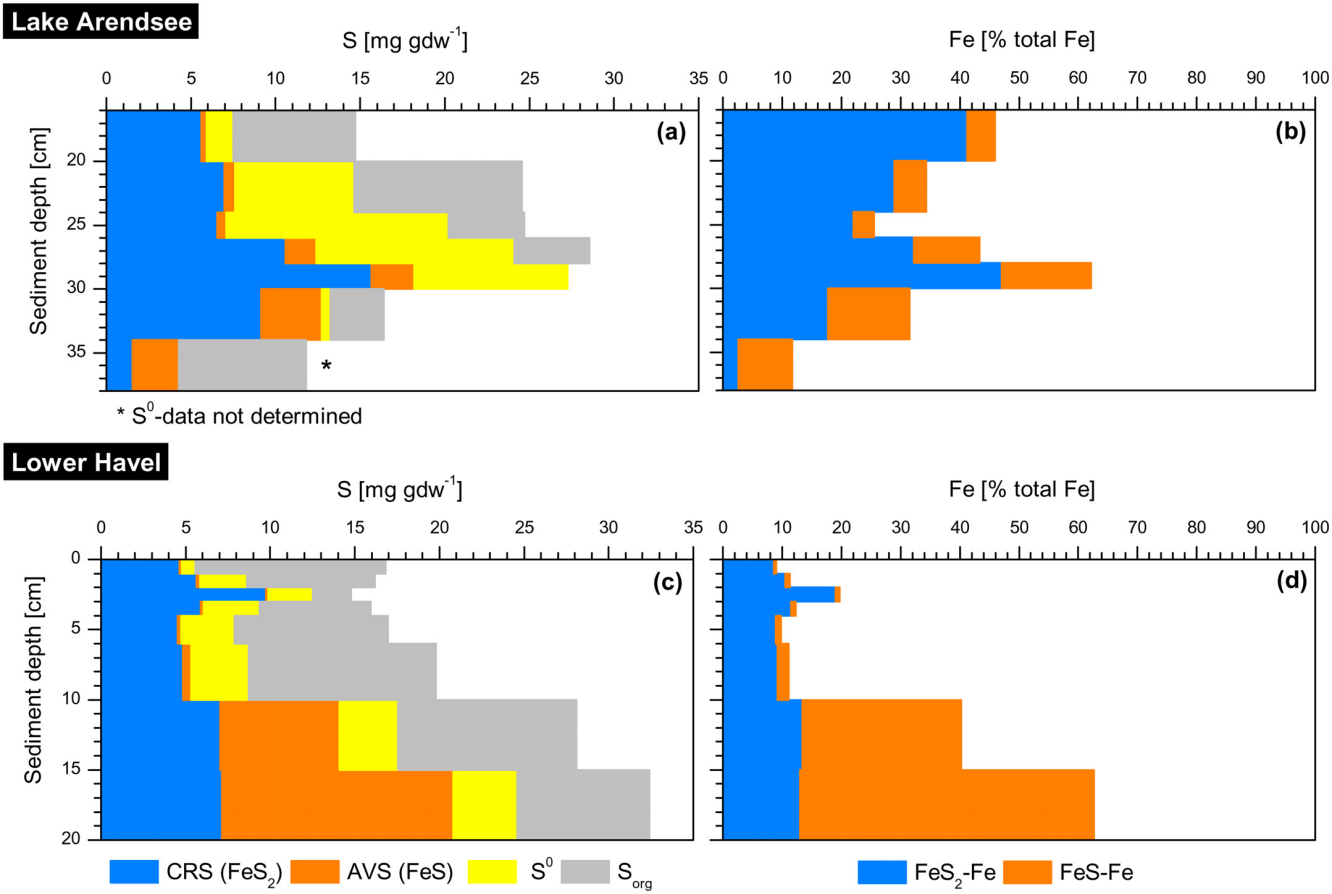
Vertical distribution of inorganic sulphur (S) species in sediments of Lake Arendsee (October 2014) and Lower Havel (May 2012). **(a, c)** CRS: chromium reducible S, AVS: acid volatile S, S^0^: elemental S and organic S (S_*org*_) in mg gdw^−1^. **(b, d)** Relative contribution of mono- and disulphidic bound Fe to the total Fe content (calculated). Note that the S peak was 10 cm further down-core here, than evident from Fig 1b. Data from Lower Havel are reprinted from [25] under a CC BY license, with permission from Springer, original copyright 2015.

In Lake Arendsee, there was a S peak in 19 cm depth (Fig 1b). In the core used for TRIS analysis this S peak was 10 cm further down-core than evident from Fig 1b. Accordingly, the highest S content was at 29 cm sediment depth, here (Fig 2a). The stratigraphy of OM and Fe had similar vertical shifts among all cores taken.

The peak in S was due to elevated content of mono- and disulphidic bound S as well as S^0^ (Fig 2a). The content of S^0^ was 10 times higher above than below the S peak. Assuming all acid volatile S (AVS) to be present as FeS and all chromium reducible S (CRS) to be present as FeS_2_, the percentage of sulphidic-bound Fe on total Fe (degree of sulphidisation) varied between 10% and 60% (Fig 2b). Within the S peak the immobilisation of Fe through the formation of Fe sulphides (FeS_x_) was highest. Below the S peak, the percentage of Fe bound to sulphides on total Fe decreased which was not only due to lower content of mono- and disulphidic bound S but also due to an increase of total Fe.

In Lower Havel, elevated concentrations of mono- and disulphidic-bound S (AVS, CRS) were present at 10–20 cm depth (Fig 2c). The increase in AVS and CRS explained the elevated content of total S below 10 cm depth. S^0^ and S_org_ did not change in the upper 20 cm. The immobilisation of Fe through the formation of FeS_x_ averaged to 12% of total Fe at 0–10 cm depth. This percentage increased to 40% at 10–15 cm depth and 63% at 15–20 cm depth (Fig 2d).

### Qualitative and quantitative vivianite analysis

**Fig 3:**
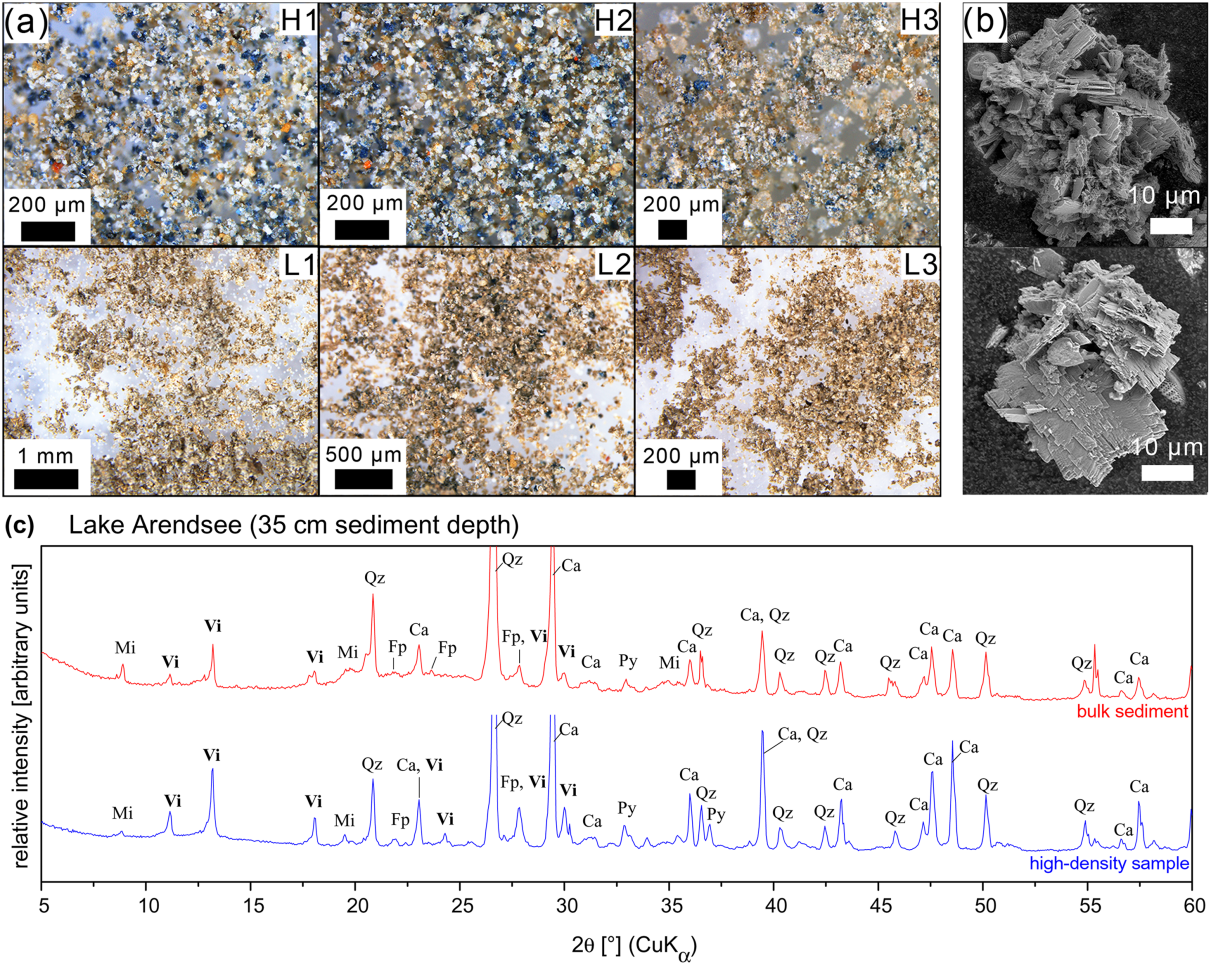
Analysis of high-density sediment samples of Lake Arendsee. **(a)** Reflected-light microscope images of high-density (*ρ* > 2.3 g cm^−3^, H1–H3) and low density (*ρ* < 2.3 g cm^−3^, L1–L3) samples from different sediment depths (positive downward sampling depth) of Lake Arendsee (H1, L1: 27 cm; H2, L2: 35 cm; H3, L3: 41 cm). **(b)** Scanning electron micrographs of dark blue vivianite nodules enriched in high-density samples. **(c)** XRD patterns of bulk sediment (data in red) and high-density samples (data in blue) from 35 cm sediment depth of Lake Arendsee. The pattern confirm the presence of vivianite (Vi). Other minerals identified were calcite (Ca), quartz (Qz), pyrite (Py), mica (Mi) and plagioclase (Fp). For clarity main peaks of calcite and quartz are not shown in total.

**Fig 4:**
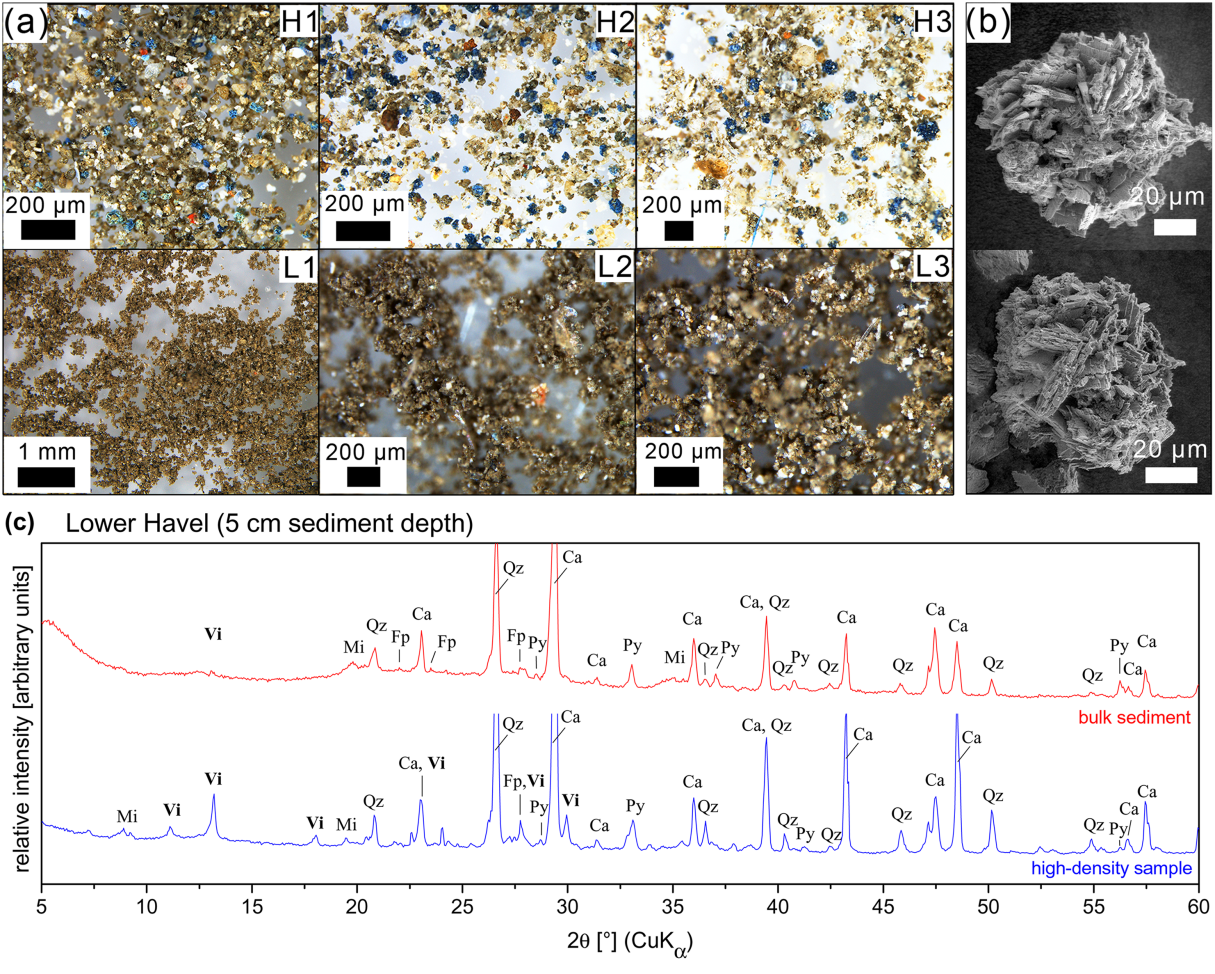
Analysis of high-density sediment samples of Lower Havel. **(a)** Reflected-light microscope images of high-density (*ρ* > 2.3 g cm^−3^, H1–H3) and low density (*ρ* < 2.3 g cm^−3^, L1–L3) samples from different sediment depths (positive downward sampling depth) of Lower Havel (H1, L1: 0.5 cm; H2, L2: 5 cm; H3, L3: 9 cm). **(b)** Scanning electron micrographs of dark blue vivianite nodules enriched in high-density samples. **(c)** XRD patterns of bulk sediment (data in red) and high-density samples (data in blue) from 5 cm sediment depth of Lower Havel. The pattern confirm the presence of vivianite (Vi). Other minerals identified were calcite (Ca), quartz (Qz), pyrite (Py), mica (Mi) and plagioclase (Fp). For clarity main peaks of calcite and quartz are not shown in total.

In both waters, i.e. Lake Arendsee and Lower Havel, dark-blue nodules were found in the high-density samples (*ρ* > 2.3 g cm^−3^), but not in the low-density samples (*ρ* ≤ 2.3 g cm^−3^) using a reflected-light microscope. These nodules were identified as the Fe(II)-phosphate mineral vivianite (Figs 3 and 4). Powder X-ray diffraction pattern showed characteristic reflexes of vivianite whereas in high-density samples without any dark blue nodules vivianite reflexes were missing. The diffraction pattern further confirmed the presence of pyrite, quartz, calcite, mica and plagioclase (Figs 3c and 4c).

In Lake Arendsee, vivianite nodules were detected at 26 cm and further down-core up to 48 cm depth. Highest numbers of vivianite nodules were present between 30 cm and 36 cm depth (Fig 3a). In these layers vivianite had such a high content that it was possible to detect the mineral in bulk sediment using X-ray diffraction (Fig 3c). The transition between vivianite bearing and non-vivianite bearing sediment layers was sharp and already at 26 cm depth numerous small-sized (< 40 *μ*m) vivianite nodules were present. The nodules turned out to be spheroidal or irregular in shape and consisted of randomly oriented platy-shaped crystals (Fig 3b). Usually vivianite nodules were smaller than 40 *μ*m in diameter, only between 30 cm and 36 cm depth larger frustules of irregular shape (up to 150 *μ*m in diameter) were present.

High-density samples containing vivianite were characterised by a lower Ca content but had a higher Fe (on average 3.7 times higher), Mn (on average 15 times higher) and P (on average 48 times higher) content compared to samples without vivianite (Table 1). The S content was similar for all samples, except for 24–26 cm and 36–38 cm depth, where S content was 7- and 10-times higher, respectively. According to the mass of each high-density sample the amount of P present in these samples accounted for 13.1±0.4% of TP in bulk sediment in layers without vivianite and 45.7±19.9% of TP in bulk sediment in layers where vivianite was present. The amount of Fe detected in high-density samples accounted for 25% and 40% of total Fe in samples with and without vivianite, respectively.

**Table 1:** Elemental composition and vivianite occurrence of high-density samples (*ρ* > 2.3 g cm^−3^) from different sediment depths of Lake Arendsee (February 2014) and Lower Havel (October 2013). The P content of high-density samples (P) is also given as percentage of total P of bulk sediment (P_Sed_).

Sediment depth [cm]	vivianite occurrence	Ca	Fe	Mn [mg gdw^−1^]	P	S	P [% P_Sed_]
***Lake Arendsee:*** **vivianite present at 26–48 cm depth**							
4–6	-	325	1.9	0.3	0.3	2.8	13
8–10	-	339	1.7	0.3	0.1	1.9	13
24–26	-	296	25.9	0.5	0.3	24.9	13
no vivianite present (mean ± SD)		320 ± 22	9.8 ± 13.9	0.4 ± 0.1	0.2 ± 0.1	9.9 ± 13.0	13.1 ± 0.4
26–28	+++	102	44.0	8.3	11.5	2.1	86
28–30	+++	126	35.0	4.2	10.8	3.2	36
32–34	+++	77	33.6	4.6	9.5	2.5	30
34–36	+++	58	42.7	9.1	12.9	1.3	50
36–38	+++	266	33.8	2.5	5.9	16.3	28
38–40	+++	105	35.6	7.6	9.1	2.2	52
40–42	++	111	35.8	9.0	7.9	2.4	39
vivianite present (mean ± SD)		121 ± 68	37.2 ± 4.3	6.4 ± 2.6	9.7 ± 2.3	4.3 ± 5.3	45.7 ± 19.9
***Lower Havel:*** **vivianite present at 0–18 cm depth**							
0–1	++	245	22.1	1.3	3.1	8.0	7.9
1–2	++	273	22.8	1.5	3.6	8.0	9.8
2–4	++	250	21.8	1.6	3.9	7.1	9.2
4–6	++	264	23.9	2.2	5.5	6.7	12.5
8–10	++	276	23.0	1.7	3.8	8.4	14.3
layers with main vivianite presence (mean ± SD)		262 ± 14	22.7 ± 0.8	1.7 ± 0.4	4.0 ± 0.1	7.7 ± 0.7	10.7 ± 2.6
12–14	+	271	18.6	0.7	1.0	11.2	6.7
16–18	+	283	19.9	0.7	1.5	15.8	9.6
18–20	-	296	22.3	0.6	0.7	32.7	7.3
20–22	-	278	22.3	0.5	0.6	26.4	4.9
no (or hardly any) vivianite present (mean ± SD)		282 ± 11	20.8 ± 1.8	0.63 ± 0.10	0.95 ± 0.40	21.5 ± 9.8	7.1 ± 1.9

number of vivianite nodules: +++ high, ++ medium, + low, - none

In Lower Havel, vivianite nodules were present in the uppermost sediment layer down to 18 cm depth. Highest numbers of vivianite nodules were present between 2 cm and 10 cm depth, but the number was considerably lower than in sediments of Lake Arendsee (Fig 4a). Between 10 cm and 18 cm depth only some single vivianite crystal aggregates were detected in the high-density samples, and further down-core vivianite was not present. Vivianite nodules with a large size were much more abundant than small nodules (< 40 *μ*m) but the overall number of nodules was lower than in samples from Lake Arendsee. Vivianite crystal aggregates had a uniform spherical shape with diameters between 40 *μ*m and 100 *μ*m and consisted of randomly oriented platy-shaped crystals (Fig 4b).

High-density samples containing numerous vivianite nodules (0–10 cm depth) had a higher Mn and P content (on average 3- and 6-times higher) than samples without vivianite, whereas Ca and Fe content was similar for all samples (Table 1). Sediment layers with vivianite nodules had a two times lower S content than samples from further down core, where only a few or no vivianite crystal aggregates were present. The P concentration of high-density samples containing vivianite accounted for 10.7±2.6% of sedimentary TP. For high-density samples without vivianite, the amount of P present accounted for 7.1±1.9% of TP. The Fe content of high-density samples did not show differences between vivianite bearing and non-vivianite bearing samples and accounted for 45.3±3.2% of total Fe.

### Phosphorus fractionation of vivianite

Two different sequential P fractionation schemes [30, 32] were carried out, in order to study the extraction characteristics of vivianite. Following [30], the synthetic mineral is extracted by two sequential steps: 14% of vivianite-P was extracted as redox sensitive Fe-bound P (BD-TP) and 84% as metal-bound P (NaOH-SRP) (Table 2). The remaining P forms accounted for less than 2.5% of TP. A similar result was obtained (28% BD-TP, 66% NaOH-SRP) when extracting a high-density sample that was naturally rich in vivianite nodules. Only a minor amount of P was extracted as loosely adsorbed P (NH_4_Cl-TP).

**Table 2:** Proportion of sedimentary phosphorus (P) forms according to [30] on total P of synthetic vivianite powder (P_viv_, surface oxidised, blue appearance) and of a high-density sample (*ρ* > 2.3 g cm^−3^) naturally rich in sedimentary vivianite. The first two steps of the P speciation according to [32] are given in brackets.

NH_4_Cl-TP (Ex-P)	BD-TP (CDB-P)	NaOH-SRP	NaOH-NRP	HCl-TP	Res-P	Recovery rate
[% P_viv_]						
***synthetic vivianite***						
0.8 (0.2)	13.8 (15.8)	83.9	1.5	0	0	93
***high-density sample naturally rich in sedimentary vivianite***						
4.0	28.2	66.3	1	0.5	0	102

**P speciation according to [30]:** NH_4_Cl-TP: loosely adsorbed P; BD-TP: redox sensitive P, mainly bound to Fe-(hydr)oxides; NaOH-SRP: metal-bound P, mainly associated with Fe- and Al-oxides; NaOH-NRP: organic-bound P, HCl-TP: P bound in calcium carbonates and apatite; and Res-P: residual P determined after digestion of remaining sediment.

**P speciation according to [32]:** Ex-P: loosely sorbed P; CDB-P: iron(oxyhydr)oxide-bound P, vivianite.

Following the P fractionation of [32] only about 16% of TP was extracted during CDB extraction which is supposed to represent iron(oxyhydr)oxide-bound P and vivianite-P [14] (Table 2).

## Discussion

### Heavy-liquid separation advances the identification and quantification of sedimentary vivianite

Sedimentary vivianite can be directly identified by application of a heavy-liquid separation, as first described by [19]. The enrichment of vivianite nodules in high-density samples enables vivianite identification by X-ray diffraction (Figs 3 and 4), and allows to evaluate the relative contribution of vivianite to sedimentary TP [19]. For both Lake Arendsee and Lower Havel, high-density samples with high P content had the lowest molar Fe:P ratios. They were as low as 1.8 (Lake Arendsee) and 2.5 (Lower Havel) which is close to the Fe:P ratio of pure vivianite (Fe:P = 1.5) indicating a high percentage of Fe-P phases in these samples. In contrast, high-density samples without any vivianite nodules, low P and high S content had molar Fe:P ratios larger than 18 indicating elevated contents of sulphidic-bound Fe which is not being able to bind P (Table 1).

Considering the mass of each high-density sample relative to the mass of the sample prior to heavy-liquid separation, the corresponding P content gives an upper limit of the amount of vivianite present in the sample. However, P compounds other than vivianite, i.e. P associated with calcium carbonates and P sorbed onto the surface of iron(oxyhydr)oxides, likely contribute to the P present in the high-density samples.

In contrast, sequential P extraction of vivianite (surface oxidised synthetic vivianite powder and naturally-born vivianite (Table 2)) demonstrates that chemical P fractionation methods are not suitable to distinguish between iron(oxyhydr)oxide-bound P and Fe(II)-phosphate minerals, even though they are a widely used tool for the characterisation of P binding forms in soils and sediments [35]. Our analysis was performed using a highly crystalline vivianite (as inferred by line width in the XRD pattern), which did not liberate P during a single extraction step, contrary to observations with freshly precipitated vivianite [36, 37]. Our results suggest that results from the SEDEX extraction scheme typically used for quantifying Fe-bound P (citrate-dithionite-bicarbonate extraction, CDB-P) may vary depending on the proportion of crystalline vivianite in natural sediments. Differences in crystallinity may explain why naturally-born vivianite is liberated to a higher extent than the synthetic vivianite analysed here in the redox sensitive Fe-bound P fraction (Table 2). Since Fe(II)-P minerals are stable under reducing sedimentary conditions, an entire liberation of these minerals after addition of a reductant is unlikely. However, both citrate and bicarbonate act as a chelating agent for Fe which can explain a variable liberation of P depending on the crystallinity of the mineral. Our results demonstrate that if freshly-precipitated, less-crystalline vivianite constitutes the predominant fraction in the sediment during sampling then the pool of potentially mobile P (BD-P, CDB-P) may be overestimated.

### Occurrence of vivianite is influenced by lake-specific environmental conditions

Most striking in our results, in Lake Arendsee, vivianite was present in deeper sediment horizons (Fig 3) and not in the uppermost layers with a sharp transition between vivianite and non-vivianite bearing layers (Table 1). In contrast, in Lower Havel vivianite was present in the upper sediment layers and not in deeper horizons (Fig 4) with a gradual transition between non-vivianite and vivianite bearing layers (Table 1). What are the reasons for the observed pattern in both waters?

In Lake Arendsee the presence of vivianite below 26 cm depth corresponds to a time prior to high anthropogenic nutrient loadings, and the abrupt shift in Fe and P content at the transition from vivianite bearing to non-vivianite bearing layers dates back to the end of the 1930’s (Fig 1c). At this time the demand on the lake and its catchment changed and the input of nutrients increased. Due to a long hydraulic residence time of 50–60 yr [22] and rising nutrient levels, primary production and hence OM supply toward the sediment increased. This led to an increased demand for oxidants (i.e., O_2_, Fe^3+^, SO_4_^2-^), deteriorating redox conditions and enhanced S^2-^ production through decomposition of OM, specifically putrefaction of organic S (desulphuration) and dissimilatory sulphate reduction (desulphurication). Subsequently, more sedimentary Fe was immobilised by the reaction with S^2-^ and the P binding capacity of the sediment was weakened.

Based upon algal microfossil facies [23] Lake Arendsee has been eutrophic since the 1940’s, but oligo-mesotrophic before that time. Vivianite was present in layers dated back to the beginning of the 20th century. This is in accordance with results from [23] who reported high P and Fe content and elevated P accumulation rates at a similar time. This observation raises the question where the high amounts of P were originating from in such a low-productive system at this time. What could be the reason for the massive decline in Fe content? Since Lake Arendsee is a karst lake, the intrusion of water rich in P and Fe [23] might explain the high amounts of P and Fe in the sediment and the occurrence of vivianite. The decline in Fe content could also be related to the disconnection of a peatland due to melioration measures close to Lake Arendsee in the 1960’s.

In Lower Havel, vivianite nodules were detected in the uppermost centimetre of the sediment, indicating that vivianite formation is an ongoing process, here. However, in deeper sediment layers (below 18 cm depth) vivianite was not present. Compared to Lake Arendsee, in Lower Havel the transition between sediment layers containing numerous vivianite nodules and non-vivianite bearing sediment layers was more gradual which was also represented in the change of the TP content with depth (Fig 1a and 1d). In this transition zone, only a very limited number of vivianite crystal aggregates were detected.

The decrease in vivianite abundance and the absence of vivianite at greater depths may be due to dissolution of the mineral through enhanced sediment sulfidisation. However, vivianite dissolution is unlikely, here. This is because in Lower Havel oxygen penetration is limited to the uppermost millimetres of the sediment and the zone of SO_4_^2-^ reduction is located just below the oxic-anoxic boundary. A pore water study from an organic-rich sediment similar to that of Lower Havel [19] showed that SO_4_^2-^ is readily depleted within in the upper 5–7 cm of the sediment. However, in Lower Havel vivianite abundance was highest in the upper 10 cm which matches with the zone where SO_4_^2-^ reduction is supposed to be highest. Notably, any vivianite crystal aggregates, neither from the upper 10 cm nor from further down-core, showed signs of pitting from a potential dissolution. Thus, the occurrence of vivianite and the increased P binding capacity of the sediment in the upper 18 cm rather indicate a change of the geochemical conditions in Lower Havel during the last 20 yr but it not reflects the dissolution of vivianite at greater depths (see explanation below).

The observed changes in P binding and the occurrence of vivianite correspond to a time between 1994 and 2006 and were accompanied by a decline in S content (Fig 1). The decline in S burial can be mainly attributed to a decline in OM production observed in Lower Havel during the last 25 yr [25]. A decrease in productivity leads to both a decline in S sedimentation by settling seston and lower sulphate reduction rates and explains the increased P binding capacity of the sediment and the occurrence of vivianite. The direct effect of changes in water column SO_4_^2-^ concentration on S burial is low because SO_4_^2-^ is not limiting for phytoplankton and sulphate reducing bacteria [38–40]. Moreover, the relative retention of S on the overall water column SO_4_^2-^ imported [41] is low in such a shallow system with a water residence time of only 19 d. For example, in Lake Tegel, which is throughflown by River Havel only 3% of the S import (mainly sulphate) is retained in the sediment which is due to the short water residence time of 50–90 d [42].

**Fig 5:**
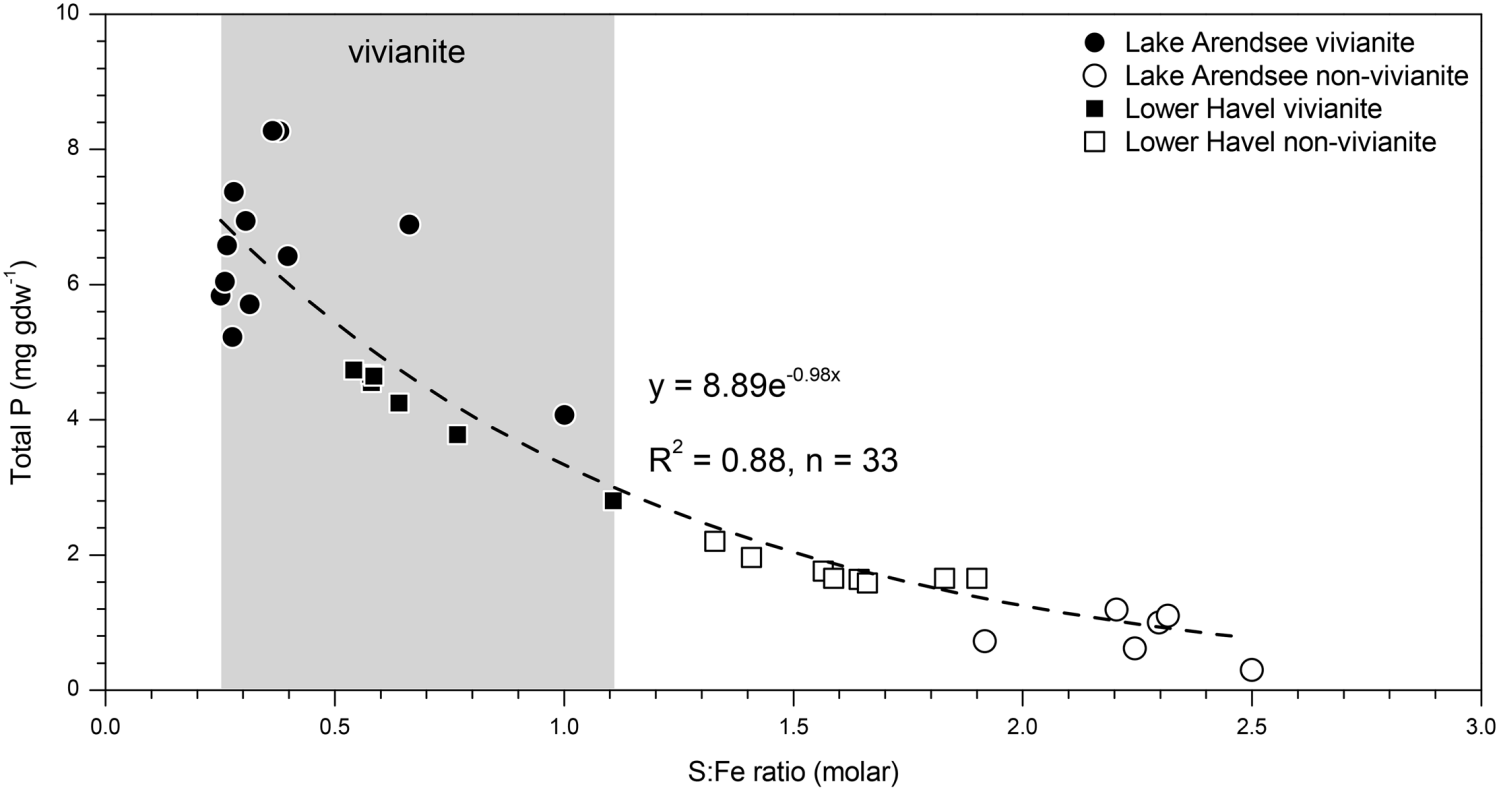
Sedimentary total P content of Lake Arendsee and Lower Havel according to the corresponding molar ratio of total sulphur to reactive iron (S:Fe) of the sediment. Vivianite occurs only at a lower S:Fe ratio.

In both waters, vivianite bearing sediment layers were characterised by elevated TP content in comparison to non-vivianite bearing sediments (Table 1, Fig 5). The TP co-varied with the observed number of vivianite nodules in different sediment depths: for Lake Arendsee, the sharp transisition between vivianite bearing and non-vivianite bearing sediment layers between 24 cm and 26 cm depth was reflected by a steep increase in TP down-core. The increase in TP in vivianite bearing sediment layers (according to P fractionation) was due to increased content of two P forms: redox sensitive Fe-bound P (BD-TP) and metal-bound P (NaOH-SRP) (Fig 1a and 1d). From the P fractionation of vivianite (Table 2) these two P forms are expected to be elevated if vivianite is present in sediments. Moreover, the content of NaOH-SRP in vivianite bearing sediment layers of Lake Arendsee was much higher than in those of Lower Havel (Fig 1a and 1d). This finding was in accordance with the high number of vivianite nodules detected in high-density samples compared to the number of nodules detected in Lower Havel (Figs 3a and 4a). Given the fact that vivianite diffraction peaks could be identified even in a bulk sediment sample (Fig 3c), the contribution of vivianite to overall P burial was certainly higher than in Lower Havel. Based upon the P content of high-density samples, in Lake Arendsee, vivianite-P equated to 46±20% of TP below 26 cm depth compared to 11±3% of TP at 0–10 cm depth in Lower Havel (Table 1). Our estimates of vivianite-P do not intend to give a precise determination, however, they allow to evaluate the relative contribution of vivianite to overall P burial in the studied systems. It is important to note, that vivianite cannot explain the increase in sedimentary TP alone. Due to a relative decrease of immobilised, sulphidic-bound Fe in vivianite bearing sediment layers, iron(oxyhydr)oxides which resisted reductive dissolution in the anoxic sediment [43, 44], contribute to the increase in sedimentary TP, too.

### The extent of sulphide formation controls the formation or absence of vivianite

Vivianite was identified in sediment layers which were characterised by a low molar ratio of total S to reactive Fe (S:Fe ≤1.1; henceforth denoted as “S:Fe”) and significantly contributed to an elevated P retention (Fig 5). Given our observational data, the S:Fe ratio is a viable indicator for the conditions that are important drivers behind the formation or absence of vivianite. Notably, the indicator allows to check for a potential presence of vivianite before performing any elaborate analysis.

The formation of vivianite crucially depends on the availability of the two major ions it consists of: Fe^2+^ and PO_4_^3-^. The prevailing redox conditions thereby govern the fate of both ions in the sediment. Under oxic conditions, Fe is not reduced and the PO_4_^3-^ released during the decomposition of OM is efficiently sorbed. Under anoxic conditions, ferric Fe and sulphate are used as alternative electron acceptors by heterotrophic bacteria leading to elevated production of Fe^2+^, PO_4_^3-^ and S^2-^ favouring the formation of secondary mineral phases such as vivianite and Fe sulphides (FeS_x_). Vivianite and FeS_x_ formation occur simultaneously (see Figs 3c and 4c) as long as the supply of Fe^2+^ exceeds the production of S^2-^. The molar S:Fe ratio is an indicator of the availability of S relative to that of Fe. The higher the S:Fe ratio, the higher the contribution of sulphidic-bound Fe to total Fe. Assuming that the formation of Fe monosulphide is the primary formation product between ferric Fe compounds and H_2_S,

$$2\;\text{FeO}\left(\text{OH}\right)+3\;{\text{H}}_{2}\text{S}\to \text{FeS}+{\text{S}}^{0}+4{\text{H}}_{2}\text{O},$$

[45] a molar S:Fe ratio smaller than 1.5 indicates that there is more reactive Fe available than it could be bound by S^2-^. Thus, not all reactive Fe is captured in sulphidic form and vivianite formation is favoured. In both waters, the S:Fe ratio was below that threshold and a decrease of sulphidic-bound Fe was observed in layers where vivianite was present (Figs 1 and 2). The occurrence of reduced Fe phosphates also has been shown for anoxic, organic-rich marine sediments and vivianite has been proposed to be a likely P form [12, 14]. In these systems, vivianite is expected to occur below the sulphate-methane transition. This is where redox conditions are low enough to favour the production of Fe^2+^ but S^2-^ already precipitated allowing vivianite to form [18]. Also these systems should be characterised by a molar S:Fe ratio smaller than 1.5 indicating an excess of Fe relative to the production of S^2-^ and vivianite formation should not be restricted by the supply of Fe.

Due to eutrophication, i.e. a primarily enhanced P supply, the P-binding capacity of a sediment will be exceeded leading to a higher P mobility and less or no vivianite formation. A higher productivity leads to a higher OM supply toward the sediment which has consequences for the formation of vivianite. First, there is a higher demand for oxidants leading to a deterioration of redox conditions and higher reduction rates of ferric Fe and SO_4_^2-^ [46]. Second, there is more S^2-^ produced because OM is specifically enriched in S compared to Fe (Redfield ratio: C_106_N_16_P_1_S_0.7_Fe_0.05_, [47]). Sulphides are formed by both desulphuration and dissimilatory sulphate reduction leading to a higher degree of sediment sulphidisation. The former can be quite significant in overall sedimentary hydrogen sulphide production, e.g. 5.1–53% [48]. Moreover, eutrophication is often accompanied by considerable inputs of SO_4_^2-^ leading to its higher availability and high rates of its consumption [9, 46]. Third, the OM itself can react with Fe forming a metal organic complex [49]. The higher the sedimentary S:Fe ratio, the less reactive Fe seems to be available reducing the potential of vivianite to form (Fig 5) because more Fe is bound in sulphidic form. Thus, under eutrophic conditions there is a negative feedback evolving through the enhanced supply of OM lowering the sedimentary P retention capacity due to less vivianite.

Aquatic systems naturally high in reactive Fe may compensate better for a eutrophication induced decrease in P retention than systems low in Fe. This implies, that an artificial supply of Fe to systems with a high level in OM, P and SO_4_^2-^ can be used as a successful measure of lake restoration leading to increased P retention through vivianite formation [11, 19]. To ensure a lasting effect on P burial, Fe has to be supplied in surplus compensating for the losses through FeS_x_ formation [11] and the reaction with OM [49]. At which magnitude vivianite finally forms in different types of sediments depends on multiple factors and remains to be further investigated. The formation of the mineral is also controlled by the availability of OM rich in P, the concomittant liberation of Fe^2+^ and PO_4_^3-^ into the pore voids of the sediment, the activity of microorganisms and resorption of PO_4_^3-^ onto the surface of remaining iron(oxyhydr)oxides.

## Conclusions

Vivianite was identified in surface sediments of two organic-rich freshwater sediments. The application of a heavy-liquid separation of sediment, leading to an enrichment of vivianite nodules in the high-density sediment fraction, appears to provide a reliable method to identify the mineral within the sediment matrix. The proof of vivianite in two contrasting freshwater systems and in different sediment depths, i.e. formed exclusively in the past or at present, revealed that the formation of vivianite strongly depends on the prevailing environmental conditions and is indicated by the molar ratio of total S to reactive Fe (S:Fe). Vivianite can be formed and contributes to a significantly higher P retention, as long as not all the available Fe in the sediment is converted to Fe sulphides. These conditions are met in sediments characterised by a S:Fe ratio smaller than 1.5. As a result of eutrophication, an increased supply of OM toward the sediment surface can lead to a temporarily increased retention of P and S. A longer lasting increase in OM supply of sediments favours the release of sulphides, and the formation of insoluble Fe sulphides leading to a lack of available Fe and to less or no vivianite formation. This weakening in sedimentary P retention, representing a negative feedback mechanism in terms of water quality, could be partly compensated by harmless Fe amendments.
